# Comparison of Deep-Fat Frying and Tray Drying on Ambient Storage Stability and Quality of Instant Noodles with and Without Catfish Powder

**DOI:** 10.3390/foods15060983

**Published:** 2026-03-10

**Authors:** Somwang Lekjing, Paramee Noonim, Narin Charoenphun, Jaraslak Pechwang, Jessada Rattanawut, Thanamat Paongoen, Karthikeyan Venkatachalam

**Affiliations:** 1 Faculty of Innovative Agriculture, Fisheries and Food, Prince of Songkla University, Surat Thani Campus, Mueang, Surat Thani 84000, Thailand; somwang.s@psu.ac.th (S.L.); parameen.n@psu.ac.th (P.N.); jaraslak.p@psu.ac.th (J.P.); jassada.r@psu.ac.th (J.R.); 6840620202@psu.ac.th (T.P.); 2 Faculty of Science and Arts, Burapha University, Chanthaburi Campus, Chanthaburi 22170, Thailand; narinch@buu.ac.th

**Keywords:** instant noodles, catfish powder, tray drying, deep-fat frying, ambient storage, lipid oxidation, microstructure, microbiological quality

## Abstract

Instant noodle fortification with fish-derived proteins enhances nutritional value; however, the effects of catfish powder (CFP) combined with different drying methods and barrier packaging on prolonged storage stability remain unknown. This study incorporated 10% (*w*/*w*) CFP into wheat flour-based instant noodles processed by tray drying or deep-fat frying, yielding four treatments: control tray-dried (CD), control fried (CF), CFP tray-dried (TD), and CFP fried (TF). Samples were packed in metallized low-density polyethylene (M-LDPE) and evaluated every 15 days over 180 days. CFP fortification increased protein and mineral content, which remained stable throughout storage. CFP incorporation and frying elevated lipid oxidation, whereas tray drying improved oxidative stability. Drying methods influenced moisture attributes, product structure, rehydration behavior, and color; tray-dried noodles retained higher lightness and hardness, whereas fried noodles showed faster water uptake. Cooking performance remained largely stable, with gradual shifts noticed in CF and TF samples over time. Microbiological quality remained acceptable, with no pathogens detected. Multivariate analysis identified the drying method as the primary driver of quality differentiation, with storage time intensifying oxidation and color divergence. Overall, tray drying with M-LDPE packaging is recommended to optimize the nutritional and storage stability of CFP-fortified instant noodles.

## 1. Introduction

Instant noodles are among the most widely consumed convenience foods worldwide, with a global demand exceeding 120 billion servings annually [1]. Low cost, ease of preparation, diverse flavor options, and extended shelf life are key factors that enhance the popularity of instant noodles. The quality attributes of instant noodles that determine consumer acceptance include texture, rehydration behavior, color, flavor, and the absence of rancid or off-flavor during storage. Most instant noodles are manufactured by steaming, followed by deep-fat frying [2]. In this process, noodles are typically fried at 140–160 °C for 1–2 min, which reduces moisture to approximately 2–5% and generates a porous structure responsible for rapid rehydration and the characteristic mouthfeel of the noodles [3]. However, steamed-and-fried instant noodles generally contain a substantial lipid fraction (typically 15–22% fat), which is considerably higher than that in hot-air-dried products (<3% fat), and the oxidative rancidity of the lipid fraction is a major factor limiting shelf life and nutritional quality [4]. Prolonged exposure of frying oils to high temperatures accelerates lipid oxidation, hydrolysis, isomerization, and polymerization, leading to the formation of primary and secondary oxidation products. In starch-rich systems, high-temperature processing can accelerate reactions that promote off-flavors, discoloration, and textural deterioration during storage at ambient temperatures [5]. Consequently, there is growing interest in alternative drying technologies and formulation strategies that can reduce fat content and improve oxidative stability without compromising sensory quality or microbiological safety [6].

From a nutritional perspective, conventional instant noodles are typically characterized by high carbohydrate content and relatively low protein and micronutrient content [7]. Therefore, partial substitution of wheat flour (WF) with fish-derived ingredients has emerged as a strategy to enhance the nutritional value of noodle products [8]. Previous studies have shown that incorporating fish meat powder, fish protein powder, or fish protein hydrolysates into noodles and pasta can increase protein content, improve amino acid balance, and elevate the levels of minerals such as calcium, iron, zinc, and long-chain n-3 polyunsaturated fatty acids (PUFAs) [9,10]. Fish-enriched instant noodles have been reported to have higher protein, omega-3 fatty acid, and micronutrient densities than conventional products, while maintaining acceptable cooking and sensory characteristics when formulation and processing are properly optimized [8,11]. Among the available fish resources, catfish is an attractive raw material for value-added cereal–fish quality proteins and lipids, including appreciable amounts of eicosapentaenoic acid (EPA) and docosahexaenoic acid (DHA), together with essential micronutrients such as selenium and zinc [12]. Therefore, utilizing CFP in instant noodle formulations can reduce the proportion of WF, enhance protein and mineral content, and introduce health-promoting long-chain n-3 PUFAs. Simultaneously, converting low-value or underutilized catfish materials into stable ingredients for noodles aligns with circular economy principles by valorizing fish by-products and improving resource efficiency in aquaculture and fish processing chains [13,14]. Accordingly, the CFP-fortified formulation is intended for consumers seeking animal-protein enrichment and is not positioned as a vegetarian or plant-based alternative.

Despite these advantages, integrating lipid-rich fish ingredients, particularly edible catfish flesh (including skin) that retains endogenous lipids rich in unsaturated fatty acids, into a cereal-based instant noodle matrix poses technological and stability challenges owing to increased susceptibility to lipid oxidation during storage. The presence of unsaturated fish lipids increases susceptibility to oxidation, flavor deterioration, and structural weakening during ambient storage [8,15]. During deep-fat frying, endogenous lipids from fish and exogenous frying oil are simultaneously exposed to high temperatures and oxygen, triggering complex chains of free radical reactions that generate hydroperoxides, aldehydes, ketones, and polymerized triacylglycerols [16]. Oxidative and Maillard reactions may continue, albeit at a slower rate, during storage, potentially compromising flavor, nutritional quality, and safety [17,18]. Consequently, understanding how processing conditions and formulation (with or without fish) influence oxidative stability, microstructural integrity, and microbiological quality is essential for developing fish-enriched instant noodles with an acceptable shelf life.

To address the health and stability concerns associated with deep-fat frying, alternative drying methods such as hot-air and tray drying have been proposed as non-fried processing routes for instant noodles [6]. In these methods, noodles are steamed and dried in hot air, leading to lower fat content (<3% and ~70% reduction vs. fried products), but with longer drying times, firmer textures, and higher cooking loss. Drying conditions (temperature, air velocity, relative humidity, and residence time) strongly affect the extent of starch gelatinization, protein denaturation, and pore formation, which in turn govern the rehydration behavior, textural attributes, and overall physicochemical quality of the finished noodles [19]. In products containing both starch and lipid fractions, such as fish-enriched noodles, drying-induced microstructural changes can modulate the accessibility of lipids to oxygen and pro-oxidant components, thereby influencing oxidative stability and the evolution of quality attributes during storage [20]. Although several studies have examined air-dried instant noodles or fish-enriched pasta and noodles, most have focused on formulation optimization or short-term quality evaluation and have not systematically compared deep-fat frying and tray drying for fish-enriched instant noodles under ambient storage for extended periods [6,21]. In particular, there is limited information on how these drying technologies affect the long-term evolution of physicochemical properties, structural characteristics, and microbial stability in CFP-enriched instant noodles packed in high-barrier packaging [22].

Consequently, there is limited evidence on how drying technology affects the quality trajectory, oxidative stability, and microbial quality of CFP-fortified instant noodles during extended ambient storage. Addressing this limitation is necessary for designing value-added, nutritionally improved, and shelf-stable noodle products. The present study comparatively evaluated deep-fat frying and tray drying as the final processing operations for instant noodles formulated with or without CFP and packed in M-LDPE packaging. This study effectively examined time-dependent changes in physicochemical attributes (proximate composition, color, texture, and related properties), structural organization, and microbial quality during prolonged ambient storage. By clarifying the combined effects of drying method, fish enrichment, and storage time, this study provides an evidence base for selecting processing strategies that support oxidative stability and shelf reliability in nutritionally enhanced instant noodles.

## 2. Materials and Methods

### 2.1. Raw Materials, Chemicals and Reagents

Catfish (*Clarias macrocephalus* × *Clarias gariepinus*, 500–800 g/fish) were purchased in a dead and fresh condition from a local fish producer in Surat Thani Province, Thailand. The fish were transported to the laboratory in an icebox within two hours of collection, washed thoroughly with clean tap water, and carefully stripped of all non-edible parts, including the head, scales, fins, tails, viscera, and bones, while the skin was retained as part of the edible portion. The edible part of the fish was washed again, cut into small pieces (approximately 4 cm long), and soaked in saline solution (4% *w*/*v* NaCl) for 30 min. After soaking, the fish pieces were allowed to rest at room temperature for 10 min before further processing. The fish samples were minced using a lab-scale mincer (TC 12 E; ITALVER SRL, Padova (PD), Italy) and boiled for 30 min. Any oil floating on the surface during boiling was manually separated before cooling the product to room temperature. Subsequently, the minced fish were passed through a cheesecloth to eliminate excess moisture. Cooked fish samples were collected and dried in a tray dryer (Model CD-9, JST Engineering Co., Ltd., Bangkok, Thailand) at 120 °C for approximately 2 h or until a constant weight was achieved (two consecutive weighings at 30 min intervals showed a difference of <0.1 g). The final moisture content of the dried catfish powder was 5% (w.b.). The dried fish was milled using a ball mill (PM 100, Retsch GmbH, Haan, Germany) and sieved through a 250 µm mesh to obtain a uniform fine powder. The final product was stored in airtight polypropylene bags at room temperature until use.

All other ingredients used for making instant noodles were purchased from local suppliers in Surat Thani Province. Analytical-grade chemicals and reagents used for physicochemical and lipid oxidation analyses included sodium chloride (NaCl), sodium bicarbonate (NaHCO_3_), citric acid, ethanol (95%), sodium hydroxide (NaOH), phenolphthalein indicator, glacial acetic acid, chloroform, saturated potassium iodide (KI), sodium thiosulfate (Na_2_S_2_O_3_), starch indicator, trichloroacetic acid (TCA), propyl gallate, ethylenediaminetetraacetic acid (EDTA), thiobarbituric acid (TBA), and 1,1,3,3-tetraethoxypropane (TEP). Microbiological media and reagents included buffered peptone water (BPW), Plate Count Agar (PCAg), dichloran glycerol agar (DGg 18), lauryl sulfate tryptose (LST) broth, E. coli (EC) broth/medium, Rappaport–Vassiliadis (RV) medium, tetrathionate (TT) broth, xylose lysine deoxycholate (XLD) agar, Hektoen enteric (HE) agar, Analytical Profile Index (API) 20E biochemical identification strips, *Salmonella* polyvalent O and H antisera, and lauryl tryptose (LST) broth supplemented with 4-methylumbelliferyl-β-D-glucuronide (LST-MUG), along with chromogenic agar for confirmation. Media and microbiological reagents were purchased from HiMedia (Mumbai, India). Unless otherwise specified, all other analytical-grade chemicals and reagents were obtained from Sigma-Aldrich (St. Louis, MO, USA) and Loba Chemie (Mumbai, India).

### 2.2. Instant Noodle Preparation and Storage

The instant noodle formulation used in this study was based on preliminary formulation trials and was subsequently optimized. Instant noodles were prepared using WF as the primary ingredient, which was partially substituted with CFP at a 10% replacement level. This formulation was referred to as the treatment. The formulations were composed of 90 g WF and 10 g CFP per 100 g of total flour. In addition, 10 g of salted duck egg white (SDEW), 2 g of NaCl, 1 g of NaHCO_3_, 0.1 g of citric acid, 27 g of distilled water, and 5 g of refined palm oil were added. The control (C) sample followed all the ingredients used in the treatment but was prepared using 100 g WF. Accordingly, instant noodles were prepared in four groups based on the formulation and drying method: CD (control, tray-dried), CF (control, deep-fried), TD (10% CFP, tray-dried), and TF (10% CFP, deep-fried).

All dry ingredients, including WF, CFP, NaCl, NaHCO_3_, and citric acid, were thoroughly mixed, after which SDEW, distilled water, and refined palm oil were added and mixed until homogenous. All ingredients were then slowly mixed to form dough, which was manually kneaded to achieve a consistent and crumbly texture. The dough was allowed to rest at room temperature (25 °C) for 15 min before further processing. After resting, the dough was rolled into sheets of approximately 1 mm and cut into noodle strands approximately 30 cm in length using a noodle cutter (Junxifu 160-series electric noodle maker, Yongkang City, Zhejiang, China). After cutting, the strands were coiled into round noodle blocks for subsequent steaming and dehydration. The noodles were then steamed for 10 min to gelatinize the starch and subsequently subjected to either deep-fat frying (without pre-drying) or hot-air tray drying, as described below.

Two post-steaming dehydration techniques were applied to produce instant noodles. In the first method, steamed noodle strands were cooled for 2–3 min at room temperature, shaped into round blocks, and deep-fried directly (no pre-drying step) in refined palm oil at 160 °C for 1 min using an electric fryer (Model PK-DF-635A; FFT Food Service Equipment, Pattaya, Thailand). In the second method, the noodle strands were shaped into round blocks and arranged in a single layer on trays and then dried using hot air at 80 °C for 90 min in a cabinet-type tray dryer (Model CD-9, JST Engineering Co., Ltd., Bangkok, Thailand) until the final moisture content reached below 12% (wet basis), which corresponded to approximately 10% moisture on day 0. The effective drying thickness corresponded to the strand cross-sectional thickness derived from the 1 mm sheet. Moisture was removed primarily by convection, as heated air circulated through the cabinet. After tray drying or deep-fat frying, all samples were cooled to room temperature (~25 °C) for 30 min and packed in metallized low-density polyethylene (M-LDPE) packages (15 cm × 20 cm, 50–70 µm thickness) at 100 g of noodles per package (two blocks of approximately 50 g each) and stored at ambient temperature for 180 days. Every 15 days of storage, noodles were subjected to various quality analyses as described in Section 2.3.

### 2.3. Quality Analysis

#### 2.3.1. Color Characterization and Visual Appearance

The color characteristics of instant noodles, including lightness (L*), redness to greenness (a*), and yellowness to blueness (b*), were measured using a colorimeter (UltraScan VIS, HunterLab, Reston, VA, USA) following the method described by Wang et al. [23], with some modifications. For each measurement, 5 g of the noodle sample was evenly placed at the center of a glass plate to ensure complete surface coverage and then measured at room temperature using the colorimeter, which was pre-calibrated with white and black tiles under illuminant D65 and a 10° standard observer. The total color difference (ΔE) in the samples was calculated using the following equation (Equation (1)):

(1) $$\Delta E=\sqrt{{\left(L_{S}^{*}-L_{0}^{*}\right)}^{2}+{\left(a_{S}^{*}-a_{0}^{*}\right)}^{2}+{\left(b_{S}^{*}-b_{0}^{*}\right)}^{2}}$$

where $L_{S}^{*}$ , $a_{S}^{*}$ , and $b_{S}^{*}$ represent the noodle samples, and $L_{0}^{*}$ , $a_{0}^{*}$ , and $b_{0}^{*}$ correspond to the control reference.

For visual appearance, representative photographs of uncooked and cooked noodles were captured on day 0 and day 180 using a digital camera (ZV-1, Sony Corporation, Tokyo, Japan). Samples were placed on a uniform background for image capture. Photographs were used for the qualitative visualization of changes in appearance, whereas quantitative color changes were evaluated using colorimeter-derived L*, a*, b*, and ΔE values.

#### 2.3.2. Microstructure Observation

Microstructural observations of the surface and cross-section of the tested instant noodles were performed using a scanning electron microscope (SEM) (SU3900, Hitachi High-Tech Corporation, Tokyo, Japan) in accordance with the method described by Khatkar and Kaur [24], with some modifications. Prior to the analysis, the noodle specimens were affixed to the stub using conductive carbon adhesive tape and subsequently coated with a thin layer of gold under vacuum using a sputter-coating system to enhance the surface conductivity. The prepared stubs with samples were then introduced into the SEM chamber, and the microstructure of the samples was recorded at a magnification of 30× for surface observation and 300× for cross-sectional observations, and both images were taken under an accelerating voltage of 5 kV.

#### 2.3.3. Physicochemical and Cooking Qualities

##### pH

Instant noodle samples (10 g) were homogenized with 100 mL of deionized water for 2 min. The water was then filtered using a muslin cloth, and the filtrate was measured using a pH meter (PB-11, Sartorius AG, Göttingen, Germany).

##### Water Activity

Instant noodle samples (10 g) were ground to obtain a fine powder, 2 g of the sample was placed in the sample cup, and a_w_ was measured using a dew-point moisture analyzer (AQUALAB 4TE, METER Group, Pullman, WA, USA).

##### Thickness

The thickness of the instant noodle samples was measured before and after cooking using a digital vernier caliper (Series 500, Mitutoyo Corporation, Kawasaki, Japan). For block-shaped samples, strands were gently separated prior to measurement, and the thickness was measured on individual strands rather than on the compact noodle block. For uncooked noodle thickness, 10 strands (*n* = 10) were randomly selected for each treatment and kept at room temperature (~25 °C) for 30 min after removal from the package to stabilize the sample temperature and moisture condition prior to measurement. The thickness of each strand was recorded at three fixed positions (midpoint and approximately 1 cm from each end) without compressing or deforming the samples. To determine the cooked noodle thickness, the noodles were boiled until no white core was visible, rapidly rinsed with cold water for 1 min, and the surface moisture was removed with a dry paper towel. Measurements were taken within 2 min following a procedure similar to that for uncooked noodle thickness. The results were reported in millimeters (mm).

##### Rehydration Ratio

The rehydration ratio of the instant noodles was measured according to the method of Ding and Yang [25], with some modifications. Noodle samples (10 g) were placed in a bowl containing 40 mL of freshly boiled water (100 °C at the start of soaking). The samples were then stirred for 30 s and covered with a lid for 2 min to minimize heat loss. The water was not actively reheated during soaking and was allowed to cool naturally. During soaking, the samples were collected and periodically pressed to observe the disappearance of the white core in the cross-section. The point at which the white core disappeared was defined as the rehydration endpoint. The following equation was used to calculate the rehydration ratio of the samples (Equation (2)):

(2) $$RR=\frac{W_{1}}{W_{0}}$$

where W_0_ represents the initial weight of the dry noodles and W_1_ represents the weight after rehydration at the defined endpoint.

##### Optimum Cooking Time (OCT)

The OCT of the instant noodle samples was measured according to the method described by Pongpichaiudom and Songsermpong [6] with slight modifications. Briefly, 10 g of instant noodle sample was placed in a 200 mL beaker containing 100 mL of hot water maintained at 90 °C. During cooking, the sample was gently stirred to prevent strand agglomeration and to ensure uniform heating. Near the expected endpoint, noodle strands were withdrawn at short intervals and pressed between two transparent glass plates to assess the presence of a white core. OCT was defined as the time at which the white core disappeared. The results were reported as minutes (min).

##### Cooking Loss

Cooking loss was measured based on the calculated difference between the weight of the noodle samples before cooking and that of the dried residue found in the cooked water. The results were expressed as percentages.

##### Degree of Gelatinization (DG)

The DG of the tested instant noodles was determined according to the method described by Parada et al. [26] with some modifications. For analysis, instant noodles were finely ground, and 7 mg was taken and hydrated with approximately 15 µL of distilled water. The samples were then hermetically sealed in aluminum pans used in a differential scanning colorimetry (DSC), and the pans were allowed to equilibrate at room temperature for 12 h before proceeding with the DG measurement. To ensure uniform thermal equilibration and standardized partial starch gelatinization prior to scanning, the sealed pans were subjected to isothermal treatment for 60 min at a selected temperature ranging from 55 to 65 °C using DSC (DSC-7, Perkin-Elmer Co., Norwalk, CT, USA) and were then cooled to 10 °C. Subsequently, the samples were scanned against an empty pan, which was used as a reference, over a temperature range of 15–99 °C at a heating rate of 10 °C/min. The DG of the instant noodle sample was calculated from the residual gelatinization enthalpy (ΔH) obtained from DSC thermograms relative to the enthalpy of the native sample (ΔH_0_, day 0). The following formula was used to calculate the DG (Equation (3)):

(3) $$DG\left(\%\right)=\left[1-\left(\frac{\Delta H}{{\Delta H}_{0}}\right)\right]\times 100$$

#### 2.3.4. Textural Profile Analysis (TPA)

The TPA of the tested instant noodle samples was measured using a texture analyzer (CT3 10K, Brookfield Engineering Laboratories, Middleboro, MA, USA) by following the method described by Yang et al. [27] with some modifications. Instant noodle samples (25 g) were immersed in 500 mL of boiling distilled water in a pan using an induction stove, and the cooking process was stopped when no ungelatinized white core of the noodle was visible. Once the endpoint was achieved, the noodles were immediately rinsed with cold water for 1 min, and excess surface moisture was removed using a paper towel. Three strands of instant noodles were used for each sample per replication. TPA was measured using a texture analyzer under the following conditions: probe type P/36R, test mode TPA, pre-test speed of 2.0 mm s^−1^, test speed of 1.0 mm s^−1^, compression levels of 70%, automatic triggering of 5 g, and a 1 s interval between the two compression cycles. TPA parameters measured in the noodle sample are as follows: hardness (N); gumminess (N); adhesiveness (N.mm); and cohesiveness, springiness, and resilience (dimensionless).

#### 2.3.5. Proximate Composition

The proximate compositions, including crude protein, crude fat, ash, and crude fiber, of the tested instant noodles were measured in accordance with the official method of analysis [28]. For crude protein, the total nitrogen content was quantified using the Kjeldahl (KjelMaster K-375, Büchi Labortechnik AG, Flawil, Switzerland) method (991.20), and the protein content was derived by applying a nitrogen-to-protein conversion factor of 6.25. The lipid content of the samples was determined using the Soxhlet extraction (AII/16, C. Gerhardt GmbH & Co. KG, Königswinter, Germany) method (Association of Official Analytical Chemists (AOAC) method no. 922.06). The ash content was obtained by incineration using the dry-ashing protocol described in the AOAC method no. 923.03. The crude fiber content was measured based on the residue remaining after sequential acid and alkaline digestion, according to the AOAC method no. 978.10. The results for proximate composition were expressed as dry-basis percentage (% d.b.) except for moisture content, which was expressed as wet-basis percentage (% w.b.). The carbohydrate content was calculated by difference using the following equation (Equation (4)):

(4) Carbohydrate(%d.b.)=100−[Moisture(%w.b.)+Protein(%d.b.)+Fat(%d.b.)+Ash(%d.b.)+Fiber(%d.b.)]

#### 2.3.6. Lipid Oxidation Indices

##### Free Fatty Acid (FFA) Value

The FFA value of the instant noodles was measured in accordance with the American Association of Cereal Chemists (AACC) 02-02.02 method [29], with slight modifications. Approximately 5.00 g of finely ground instant noodle sample was transferred into a 250 mL Erlenmeyer flask and mixed with 50 mL of neutralized 95% ethanol solution. The mixture was gently agitated to dissolve the lipid fraction, heated to 60 °C for 10 min to facilitate extraction, and then cooled to room temperature. Phenolphthalein indicator (2–3 drops) was added, and the solution was titrated with 0.10 N NaOH until a faint pink color persisted for at least 30 s. A blank determination was performed using the same solvent and procedure without the sample.

The FFA value, expressed as mg NaOH per 100 g of sample, was calculated using Equation (5):

(5) $$FFA\;value=\frac{60\times \left(T-B\right)\times N\times 100}{Wt\times (100-M)}\times 100$$

where T (mL) is the NaOH volume for sample titration, B (mL) is the NaOH volume for blank titration, N (mol/L) is the normality of NaOH (0.10 N), Wt (g) is the sample mass (5.00 g), and M (%) is the moisture content of the sample.

##### Peroxide Value (PV) and Thiobarbituric Acid Reactive Substance (TBARS)

PV and TBARS were quantified in the instant noodle samples following the method described by Zamankhani et al. [30] with some modifications.

For PV, 5 g of noodle samples were finely ground and transferred into a 250 mL conical flask and extracted with 30 mL of a solvent composed of glacial acetic acid and chloroform (3:2, *v*/*v*). After the fat was extracted, it was mixed with 0.5 mL of saturated KI solution, and the flask was closed immediately, mixed gently, and held in the dark for 1 min. Then, 30 mL of distilled water was added, and the liberated iodine was titrated against 0.01 N Na_2_S_2_O_3_ in the presence of 1 mL of 1% starch indicator. For the blank, the same procedure was followed, except that the sample was replaced with the reagent. PV was calculated using the following equation, and the results were expressed as meq O_2_/kg of the sample (Equation (6)):

(6) $$PV=\frac{\left(V-V_{0}\right)\times N\times 1000}{m}$$

where V and V_0_ are the titration volumes (mL) for the sample and blank, respectively. where N represents the normality of Na_2_S_2_O_3_ and m represents the sample mass (g).

For TBARS, 5 g of the ground noodle sample was homogenized with 25 mL of 7.5% (*w*/*v*) TCA containing 0.1% propyl gallate and 0.1% EDTA. The homogenate was then filtered through Whatman filter paper (no. 1), and 5 mL of aliquot was collected and mixed with 5 mL of 0.02 M TBA in sealed tubes. The reaction mixture was placed in a water bath (Memmert GmbH & Co. KG, Schwabach, Germany) and heated for 30 min at 95 °C, followed by rapid cooling under running tap water. The reaction mixture was measured at 532 nm using a UV-Vis spectrophotometer (GENESYS 10S, Thermo Fisher Scientific, Madison, WI, USA). The TBARS concentration was determined from a calibration curve prepared with TEP and reported as mg malondialdehyde per kg of sample (mg MDA/kg).

#### 2.3.7. Microbial Analysis

Microbiological analysis of the instant noodle samples was conducted according to standardized ISO procedures. For each analysis, 25 g of instant noodles were aseptically weighed into a sterile stomacher bag and combined with 225 mL of sterile BPW, according to the ISO 6887 series specifications for food homogenate preparation. The mixture was homogenized using a stomacher (Seward, West Sussex, UK) to obtain a primary 10^−1^ dilution. Subsequent decimal dilutions were prepared as needed for enumeration and most probable number (MPN) determinations. For all quantitative assays, results below the method detection limit were recorded as not detected (ND) when they had a lower limit of detection (LOD). For total aerobic plate count (TPC), aerobic mesophilic bacteria were enumerated according to ISO 4833-1:2013 [31]. Appropriate dilutions were pour-plated in duplicate on Plate Count Agar and incubated at 30 ± 1 °C for 72 ± 3 h. Then, plates containing 30–300 colonies were counted, and the results were expressed as log_10_ colony-forming unit (CFU)/g. For yeast and mold, the samples were tested in accordance with the method of ISO 21527-1:2008 [32]. Appropriate dilutions were surface-plated in duplicate on dichloran glycerol (DG18) agar, which is suitable for low-moisture foods (a_mv_ ≤ 0.95), and incubated at 25 ± 1 °C for 5–7 days. Results were expressed as log_10_ CFU/g. For total coliforms, the samples were tested for coliform enumeration using the MPN method as described in ISO 4831:2006 [33]. Aliquots from the appropriate dilutions were inoculated into LST broth and incubated at 37 ± 1 °C for 24 ± 3 h. Tubes showing gas production (presumptive positive) were transferred to EC medium and incubated at 44 ± 0.5 °C for 48 ± 3 h for confirmation. MPN values were determined, and the results were expressed as MPN/g. For detection of *Salmonella* spp., samples underwent a qualitative detection of *Salmonella* by following the method of ISO 6579-1:2017 [34]. A 25 g test portion was pre-enriched in 225 mL of buffered peptone water at 37 ± 1 °C for 18 ± 2 h. Selective enrichment was conducted by transferring aliquots to Rappaport–Vassiliadis (RV) medium (incubated at 41.5 ± 1 °C) and TT broth (incubated at 37 ± 1 °C) for 24 ± 3 h. Enrichment cultures were streaked onto XLD agar and HE agar and incubated at 37 ± 1 °C for 24 h. Presumptive *Salmonella* colonies were confirmed by biochemical characterization (API 20E) and serological testing using polyvalent O and H antisera. Results were reported as detected or not detected in 25 g samples. For detection of *E. coli*, the samples underwent qualitative detection of *E. coli* using the method of ISO 7251:2005 [35]. Appropriate dilutions were inoculated into LST-MUG and incubated at 37 ± 1 °C for 24 ± 2 h. Tubes exhibiting gas production and/or fluorescence under UV light (365 nm) were considered presumptive positive and sub-cultured in *E. coli* medium for incubation at 44 ± 0.5 °C for 24 ± 2 h. Final confirmation was performed using chromogenic agar. Results were reported as detected or not detected in 25 g of sample.

### 2.4. Statistical Analysis

All experiments were performed in six replicates, and data are presented as the mean ± standard deviation (SD). The effects of formulation treatment (CD, CF, TD, TF) and storage period (0 to 180 days) on the physicochemical, textural, and color properties were analyzed using a two-way Analysis of Variance (ANOVA). When significant differences were detected (*p* < 0.05), Tukey’s Honestly Significant Difference (HSD) test was performed to separate the means. Pearson’s correlation coefficient (r) was calculated to determine the strength and direction of linear relationships between the measured variables. Additionally, Principal Component Analysis (PCA) was conducted to visualize the underlying structure of the data and to identify the variables contributing most to the variation between samples during storage. All statistical analyses were performed using SPSS (v 31.0) software (IBM Corp., Armonk, NY, USA). Figures were made using GraphPad Prism Software (v10.4.2). Pearson correlation and heatmaps were generated using TBtools (v2.3) software. PCA biplots and hierarchical cluster analysis were performed using OriginPro 2022 (v9) software. Statistical significance of tested data was established at *p* < 0.05.

## 3. Results and Discussion

### 3.1. Color Attributes

**Figure 1:**
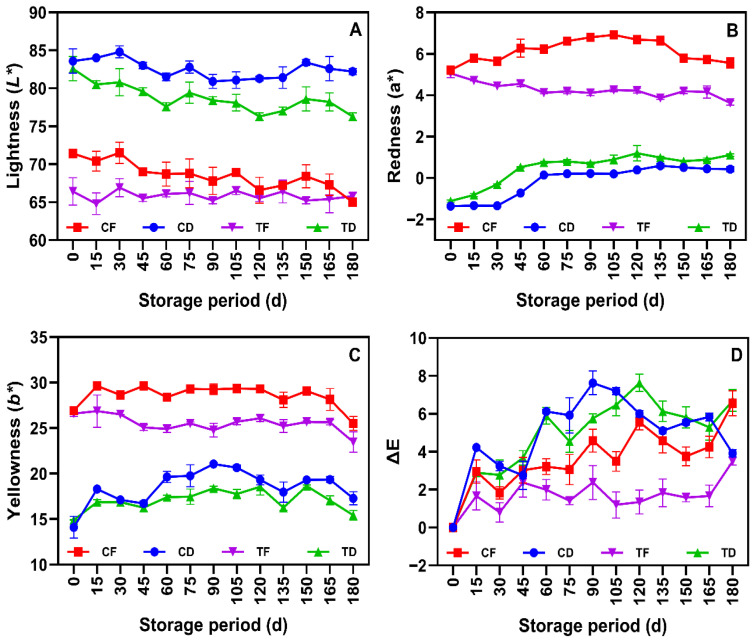
Changes in lightness (L*) (**A**), redness (a*) (**B**), yellowness (b*) (**C**), and total color difference (ΔE) (**D**) of instant noodles with or without CFP incorporation, processed by tray drying or deep frying, and stored under prolonged ambient conditions in M-LDPE packaging.

Color attributes (L*, a*, b* and ΔE) are one of the critical physicochemical attributes that influence the consumer perception of instant noodle quality [36]. Overall, the color results exhibited significant changes in the tested samples during prolonged storage at ambient conditions and the results were mainly affected by drying conditions, and with or without CFP (Figure 1A–D). Among the samples, the L* values exhibited significantly higher values in the CD and TD samples (in the range of 75–85) as compared to CF and TF (65–72) (Figure 1A). It is obvious that the substantial reduction in the L* values, especially in the deep-fried samples at the initial day of storage, could be attributed to non-enzymatic browning, primarily the Maillard reaction, where the reducing sugars from the WF reacted with the amino group from the proteins at higher temperatures, thus generating melanoidin pigments [37]. Furthermore, the addition of CFP in the noodle composition enhanced the free amino acid concentration and reactive lysine residues, which is the precursor for accelerating the Maillard reaction during the steaming and dehydration stages, leading to a darker final product. The results also found that incorporating CFP in the noodle composition had further decreased the L* values in both dried and fried sample groups. It can be seen that TD and TF samples were consistently darker than their respective controls. This phenomenon is consistent with the study of Nawas et al. [38], who tested the color characteristics of protein-enriched noodles and found that a decline in color could be attributed to two primary factors, such as ash and pigment content from the protein source. CFP contains heme pigments, particularly myoglobin and hemoglobin, which may absorb light and reduce luminosity [39]; however, because CFP is subjected to high-temperature drying during powder preparation, thermal denaturation and oxidation of these pigments likely occur primarily during CFP processing, contributing to subsequent color darkening in CFP-fortified noodles. In addition, several studies have reported that fish powder (FP) typically has a higher ash content than WF, and the elevated mineral content in FP would also be negatively correlated with L* due to the formation of insoluble mineral-phenolic complexes [40,41]. However, during the prolonged 180 days of storage, a gradual decreasing trend in L* was observed across all samples. This time-dependent darkening is likely due to the progression of the Maillard reaction even at ambient temperatures, as well as the polymerization of lipid oxidation products with proteins [42]. However, the L* results during prolonged storage exhibited a narrow effect, suggesting that a combination of low moisture level and high-barrier packaging might effectively mitigate the intensity of the darkening effects. Notably, storage time exerted a consistent influence on luminosity across all treatments. By day 180, all samples showed lower L* than day 0, confirming progressive darkening during ambient storage.

The chromatic coordinates, such as a* and b* values, revealed distinct differences during prolonged aging of tested instant noodles (Figure 1B,C). CF and TF samples displayed significantly higher b* values than the CD and TD samples. This is primarily due to the absorption of frying oil in the samples, which imparted the carotenoid-based yellow pigments to the noodle surface; however, the stability of b* values varied with time. Among the samples, TF had maintained relatively stable b* values compared to CF, which showed fluctuations. CFP-incorporated noodle samples exhibited higher stability in retaining b* values, suggesting that endogenous antioxidative constituents naturally present in fish muscle may help limit lipid oxidation and thereby reduce discoloration associated with oxidative deterioration of residual frying oil in the instant noodle matrix [43]. The a* results of the tested samples indicated a shift towards redness in the noodle samples during storage, and especially this shift was more prominent in the CF and TF samples. TF samples exhibited the highest initial a* values, and this is likely due to the oxidation of pigment, particularly myoglobin transformed into metmyoglobin in the CFP during thermal processing. Over the prolonged storage, a slight increase in a* values was observed across the samples, indicating the Strecker degradation, which is a sideline reaction of the Maillard pathway that produces red-brown pigments and aldehydes, contributing to the aged color profile of the noodles [44].

Comparison at day 180 further confirmed a sustained shift toward redness, which was more evident in the fried samples (CF and TF) than in the tray-dried samples (CD and TD). The ΔE differences in the noodle samples provide a comprehensive index of the color stability during storage (Figure 1D). A distinct divergence was observed on the ΔE values of the instant noodles that are prepared with tray-dry and deep-fry methods. Among the samples, TD exhibited poor color stability, with ΔE values rising sharply to above 6 after 90 days of storage. Unlike frying, tray drying uses a lower thermal intensity; however, because steaming/boiling and subsequent dehydration would be expected to largely inactivate endogenous enzymes, the observed discoloration during storage is more plausibly attributed to non-enzymatic mechanisms, including continued Maillard-type browning and oxidative reactions (for example, polymerization of lipid oxidation products with proteins). Furthermore, the porous structure of dried noodles allows for greater oxygen diffusion, accelerating the oxidative browning of the fish lipids present in the powder [45]. Conversely, the TF sample demonstrated superior stability and maintained a ΔE generally well below 4.0 throughout the storage period. Importantly, comparison at day 180 indicates that TD exhibited the greatest overall color deviation, whereas TF showed the highest color stability, confirming clear treatment differences in long-term color deterioration patterns. This could be due to the encapsulation effect of the frying process. Gulia et al. [21] reported that the rapid dehydration of noodles during drying creates a rigid, oil-impregnated surface crust that encapsulates the noodles and provides a barrier against oxygen and moisture. Additionally, the high-temperature processing likely generated advanced Maillard reaction products, which have been reported to possess radical-scavenging activity [46], and along with the antioxidant properties of CFP, could preserve the color integrity of the deep-fried noodles more effectively than the tray-dried noodles. Furthermore, the moderate color drifts in the noodle samples observed over a prolonged period underscore the protective efficacy of the M-LDPE packaging. LDPE- and PP-based plastic films are extensively utilized for processed fish and snack products due to their superior barrier properties against water vapor and, when metallized, against oxygen and light [47].

### 3.2. Macroscopic and Microstructural Characteristics

#### 3.2.1. Macroscopic Characteristics

**Figure 2:**
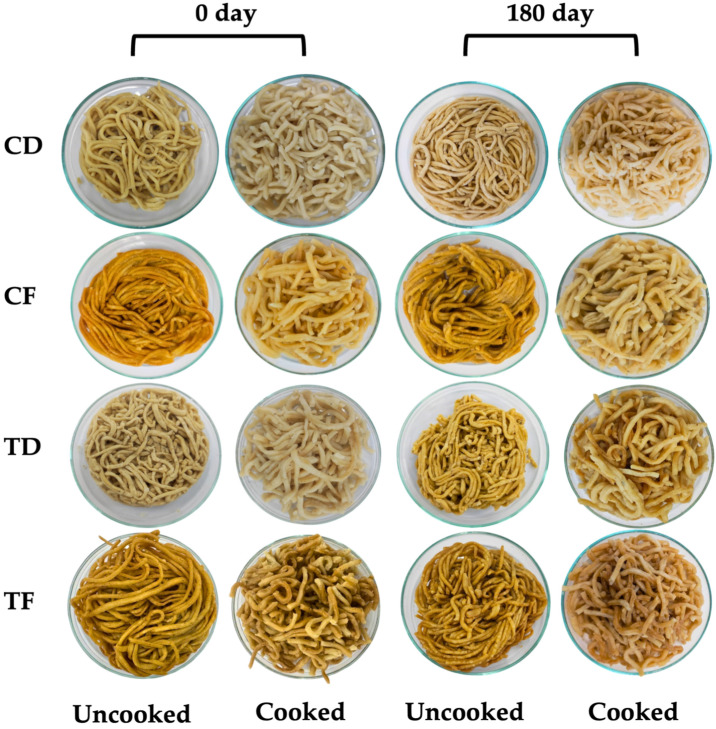
Macroscopic appearance of uncooked and cooked instant noodles with or without CFP incorporation, processed by tray drying or deep frying, and stored under prolonged ambient conditions in M-LDPE packaging (0 and 180 days).

The changes in the macroscopic appearance of instant noodles formulated with or without CFP, processed by tray drying or deep-frying, and stored under ambient conditions are shown in Figure 2. Although photographic differences among some treatments and storage time points may appear subtle, instrumental color measurements (L*, a*, b*) and ΔE are more sensitive and were used as the primary basis for evaluating color change during storage. Therefore, the photographs are provided as representative qualitative visualizations, while the numerical color data support the quantitative comparison. Processing methods strongly influence the appearance of noodle samples, exhibiting distinct visual divergence [6]. The CF and TF samples exhibited a characteristic golden yellow to reddish-brown colored surface, accompanied by a porous and blistered texture, which is typical of instant noodles [6,23]. This appearance indicates the direct results of rapid water evaporation and simultaneous non-enzymatic browning reactions (primarily Maillard reactions) at elevated temperatures, particularly deep frying [16]. Furthermore, the absorption of frying oil during preparation could also contribute to the glossy and translucent appearance of fried instant noodles [48]. In contrast, the CD and TD samples appeared opaque, pale, and comparatively dull, indicating a mild thermal effect owing to the absence of frying oil. Overall, these photographs were used to qualitatively illustrate processing-induced structural differences (porosity, surface blistering, and block integrity) and their evolution during storage. In relation to their controls, the CFP incorporation in the noodle samples showed darker and more grayish-brown characteristics, which is consistent with the introduction of additional protein, minerals, and fish-derived constituents, which could adversely influence the overall reflectance and increase the pool of Maillard reactants [49]. Additionally, prolonged storage revealed critical differences among the tested noodle samples; at day 180, changes in cooked appearance were more evident in CFP-containing samples, including reduced strand separation and a more compact cooked mass compared with day 0, suggesting storage-associated changes in noodle structure and hydration behavior.

On day 180, TF generally retained a golden-brown appearance in the uncooked state; however, the cooked TF showed a noticeable shift toward a duller, more brownish tone compared with day 0, indicating storage-associated color deterioration. These macroscopic changes were consistent with the time-dependent physicochemical changes in the noodle matrix during storage, which can influence hydration and structural integrity. Although packaging could substantially reduce light exposure and provide a better barrier against oxygen and moisture, it cannot completely eliminate the oxidative changes in the samples during prolonged storage owing to residual headspace oxygen and permeability pathways at seals [50]. Therefore, the observed darkening in CFP-containing noodles is more plausibly attributed to cumulative non-enzymatic reactions and lipid oxidation rather than enzyme-driven browning, given the steaming step used during noodle production [51,52]. In tray-dried samples, particularly TD, non-uniform darkening at 180 d may reflect similar oxidation/browning pathways, potentially promoted by structural heterogeneity and lipid exposure within the matrix. Overall, macroscopic observations indicated that frying delivered the characteristic commercial color immediately after processing, whereas CFP incorporation increased susceptibility to storage-related color drift, especially in cooked product appearance, and packaging alone was insufficient to fully prevent long-term darkening under ambient conditions.

#### 3.2.2. Microstructural Characteristics

**Figure 3:**
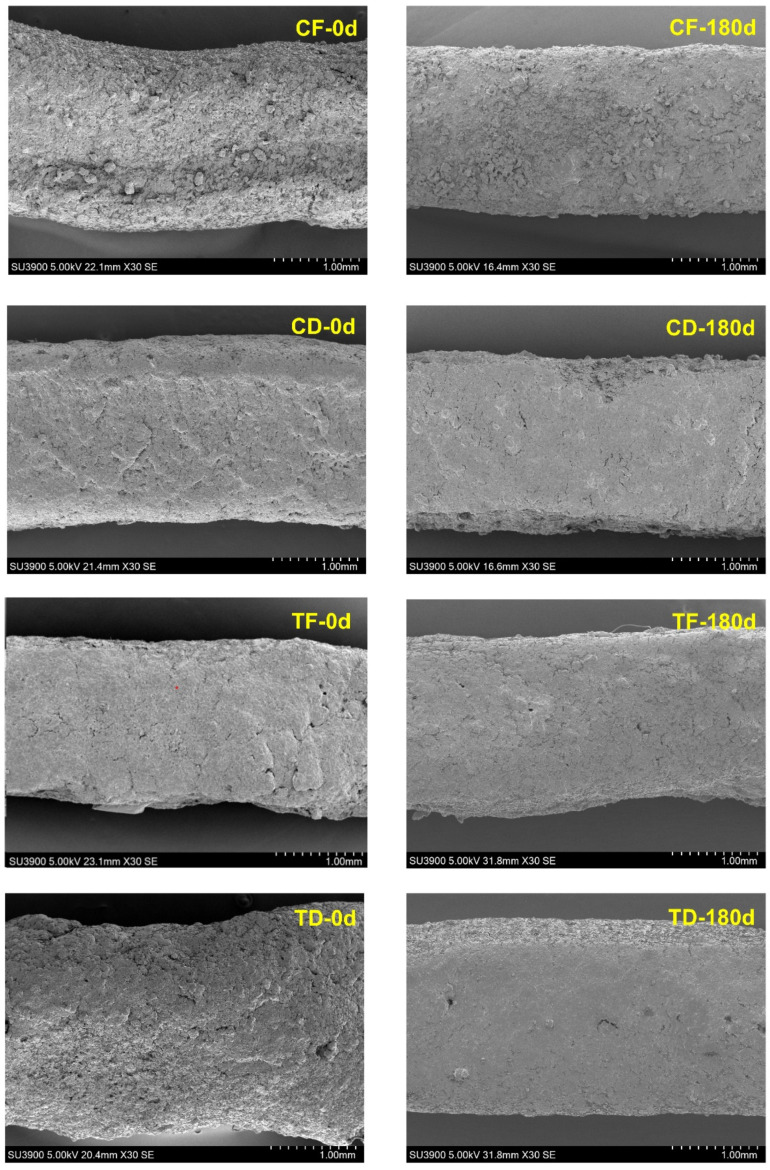
Surface section of the microstructure of instant noodles with or without CFP incorporation, processed by tray drying or deep frying, and stored under prolonged ambient conditions in M-LDPE packaging (0 and 180 days).

**Figure 4:**
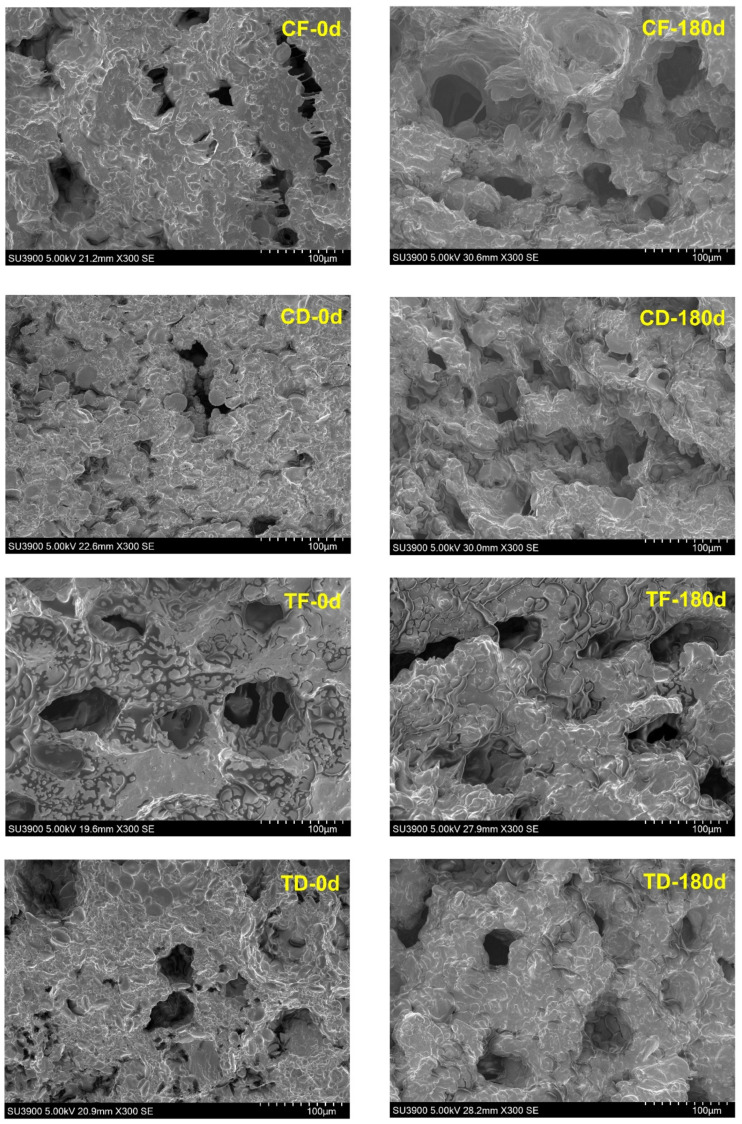
Cross section of the microstructure of instant noodles with or without CFP incorporation, processed by tray drying or deep frying, and stored under prolonged ambient conditions in M-LDPE packaging (0 and 180 days).

The microstructural characteristics of the surface and cross-section of instant noodles produced by tray drying or deep fat frying with or without CFP were tested on the initial day (day 0) and at the end of the storage day (day 180) under ambient conditions, as shown in Figure 3 and Figure 4. Overall, the SEM results demonstrated a strong influence of the drying methods, in which the fried noodles exhibited a pore-rich and blistered microstructure, and the tray-dried noodle samples exhibited a comparatively compact and more continuous matrix [6,18]. In general, deep frying removes water predominantly by rapid vaporization under a high heat flux, where internal steam generation produces vapor-pressure-driven expansion and pore formation. In contrast, tray drying removes moisture primarily by convective heat transfer and diffusion-controlled evaporation at a slower rate, with limited vapor-pressure expansion, which promotes matrix shrinkage and tighter packing of starch–protein components, thereby yielding a denser structure [53]. On day 0, the fried samples, such as CF and TF, showed a rougher and more heterogeneous surface, which is consistent with the rapid moisture vaporization that produces localized expansion, blistering, surface irregularities, and structural settling under high thermal loads, which are evident in fried instant noodles [23,54]. In contrast, the tray-dried samples, including CD and TD at day 0, appeared comparatively smoother and had a more matte surface, reflecting the absence of an intense vapor-driven surface, which generally occurs in the fried samples. In each dehydration method, the CFP incorporation also influenced the microstructure. In fried noodles, TF exhibited relatively larger and more open void regions than CF, whereas in tray-dried noodles, TD appeared more heterogeneous than CD, suggesting that CFP disrupted matrix continuity and promoted localized discontinuities. However, after 180 days of storage, all the samples showed some degree of surface consolidation, particularly with tray-dried samples, exhibiting a visibly more compact surface appearance compared with day 0. Surface consolidation indicates the formation of a more compact and rigid outer layer caused by moisture loss, matrix shrinkage, and tightening of the starch–protein network during dehydration and prolonged storage. Prolonged storage induces progressive firming, which is typical in low-moisture starch-protein foods as the matrix relaxes and reorganizes [55].

The cross-sectional observation of the tested noodle samples provided a higher mechanistic resolution of the internal structure (Figure 4). On day 0, the CF samples exhibited a honeycomb-like porous cross-sectional network with numerous irregular voids separated by thin walls. Similarly, the TF also exhibited a similar pattern but relatively larger and more open void regions. This suggests that the addition of CFP altered the wall continuity and pore geometry during frying. This is in accordance with the study of Nawaz et al. [38], who also observed that protein-enriched instant noodle formulations exhibited disrupted and more open pore networks. In contrast, the CD samples displayed a dense interior with a smaller and less open void structure than the fried noodles. TD appeared more structurally heterogeneous than the CD at day 0, and this indicates that incorporation of CFP in the noodle formulation may disrupt matrix continuity and increase local discontinuation as the particles and associated constituents are not fully integrated into the starch-gluten network. Mathew et al. [8] reported that proteins, sugars, lipids, and fibers could act as diluents and/or dispersed-phase fillers, which reduces the stretchability of the starchy matrix during noodle processing. At the end of the storage period, the fried samples retained more visible void structures, but the internal walls appeared thicker and more consolidated than at day 0, suggesting pore coalescence and/or partial collapse with matrix tightening during prolonged storage. Prolonged storage can promote structural relaxation and tightening of the low-moisture starch–protein matrix, leading to thicker pore walls and a more stable pore architecture, which can make internal features appear more clearly defined in SEM images at day 180. This structural assessment provides strong evidence for the storage-related increase in firmness, resistance to compression, and deformation. On the other hand, the CD and TD samples on day 180 showed more apparent open regions than on day 0, but the TD samples exhibited large cavities with smoother surrounding walls, indicating that prolonged storage could reorganize the microstructure of the low-moisture noodle systems. Overall, these findings suggest that subtle moisture redistribution and physical relaxation of the starch-protein matrices could alter the void distribution and water penetration pathways, thereby adversely influencing the cooking behavior of the noodles over prolonged storage [56].

### 3.3. Physicochemical and Cooking Properties

**Figure 5:**
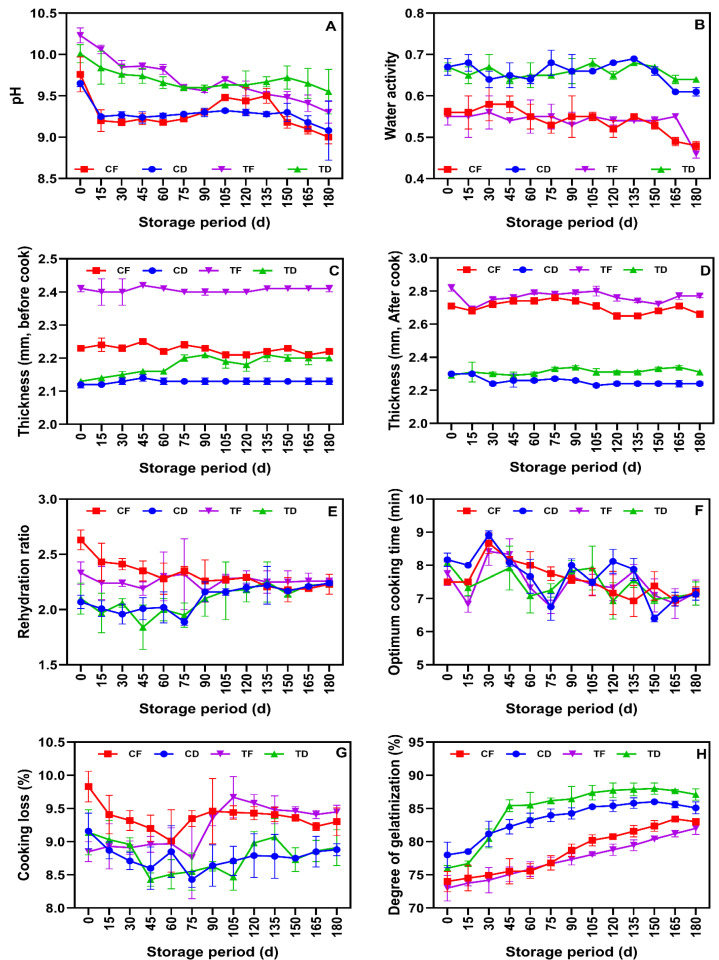
Changes in pH (**A**), water activity (**B**), thickness before cooking (**C**), thickness after cooking (**D**), rehydration ratio (**E**), optimum cooking time (**F**), cooking loss (**G**), and degree of gelatinization (**H**) of instant noodles with or without CFP incorporation, processed by tray drying or deep frying, and stored under prolonged ambient conditions in M-LDPE packaging.

The changes in the physicochemical and cooking properties of the tested instant noodle samples are shown in Figure 5A–H. The pH values of the tested instant noodle samples are shown in Figure 5A. All the tested noodle samples exhibited pH levels within the neutral to slightly alkaline range (6.3–7.5). Samples containing CFP, such as TD and TF, resulted in a slightly higher initial pH than their control counterparts due to the alkalinity of fish proteins [57]. During storage, noodle samples showed a slight drop in pH, which could be attributed to lipid oxidation, FFA formation, and Maillard reactions [58,59]. CF and TF samples showed a greater decline in pH due to their higher fat content [18]. M-LDPE packaging significantly controlled the vast changes in the pH of samples, indicating a good barrier against oxygen and moisture ingress [60]. The a_w_ values of the instant noodle samples ranged from 0.46 to 0.67, and as the storage period was prolonged, the a_w_ gradually declined; however, the changes were minimal (Figure 5B). Labuza et al. [61] reported that a decline in a_w_ in packaged low-moisture food during storage is due to the equilibrium between the product and headspace. Overall, the results showed that a_w_ in the samples was below the critical threshold (a_w_ ≤ 0.6) for microbial growth [23], indicating strong storage stability. Among the samples, fried noodles such as CF and TF had shown a lower a_w_ (0.46–0.56) than tray-dried samples (0.61–0.67). This may be due to the lower residual moisture and higher hydrophobic matrix during deep-fat frying [6]. M-LDPE packaging effectively controls vapor transmission throughout storage. Furthermore, the higher a_w_ in TF and TD is due to CFP inclusion, which is due to the hydrophilic nature of fish protein [62].

The thickness of the uncooked instant noodle strands was primarily influenced by the formulation and drying methods and remained stable with minor fluctuations during storage (Figure 5C). The lower moisture content in the noodle samples and M-LDPE packaging storage effectively preserved the strand geometry during storage, thus maintaining the stability of the noodles. Among the treatments, the uncooked TF samples showed consistently high thickness, whereas the CD samples were the thinnest. The higher thickness of the TF samples reflects the increased solid content and altered dough structure due to the incorporation of CFP, which influenced dough rheology, particularly sheet formation and strand compaction, compared to the control. Nawaz et al. [63] reported that the addition of fish meat to noodle composition induced a strong interaction between fish protein and starch and altered the rheology of the noodles. After cooking, the thickness of the tested noodle samples increased and exhibited similar trends, as the CF and TF samples retained more thickness, while the CD and TD samples were found to be low (Figure 5D). This is in alignment with the finding that frying produces a porous, sponge-like microstructure that promotes rapid water uptake and swelling during reconstitution, whereas non-fried noodles are denser and swell less under similar conditions [3,6]. The rehydration ratios of the tested noodle samples are shown in Figure 5E. Overall, the results showed that fried noodles were superior to their dried counterparts in terms of rehydration. The results confirmed that the reconstitution was higher in fried noodles, even with CFP incorporation, mainly because of the porous network, which shortens the water migration path and accelerates the hydration of the noodle core [3]. However, during prolonged storage, the rehydration ratios of the CF and TF samples declined during the mid-storage period, whereas those of the CD and TD samples gradually increased after 90 days. Because starch gelatinization occurs during steaming/cooking prior to storage, the observed changes in rehydration during storage are more plausibly attributed to storage-induced structural evolution of the low-moisture noodle matrix. In fried noodles, progressive matrix densification/compaction and rigidification of the starch–protein network, consistent with a more glassy and less permeable structure, may reduce pore connectivity and restrict water diffusion, thereby lowering water uptake during rehydration. In contrast, in tray-dried noodles, structural relaxation, moisture redistribution, and microcrack formation during storage can improve water accessibility and capillary pathways, resulting in gradually increasing rehydration after extended storage without pronounced dimensional changes [64]. The OCT of the tested noodle samples remained within 6.5–9.0 min and exhibited no consistent trend (Figure 5F). In general, the frying process typically reduces the cooling or rehydration time in instant noodle systems by increasing the porosity; however, the magnitude of the effect is dependent on the steaming conditions, strand thickness, and formulations [3,23].

This study showed no persistent separation at the optimum cooking time among the tested samples, which reflects the common steaming step that can standardize the initial degree of instantization. Consequently, the differences in the post-processing of the noodle samples were more apparent in swelling and solid retention than in the cooking endpoint. The cooking loss of the tested noodle samples is shown in Figure 5G. Cooking loss is an important indicator of solubilized solid leaching and reflects the matrix integrity of noodle samples during cooking [65]. Overall, the cooking loss remained in the range of 8.4–10% across all samples. The CD and TD samples were stable and exhibited the lowest cooking loss. The lower cooking losses in the tray-dried instant noodle samples were consistent with previous reports that well-processed air-dried noodles maintain strong microstructural continuity, which is formed during steaming and drying, whereas this is absent in fried noodles [66]. In contrast, CF was higher during the initial storage period, gradually decreased during the middle of storage, and later showed an increasing trend. TF samples peaked at mid-storage and then gradually decreased to baseline. The transient increase in TF samples during mid-storage suggests a temporary weakening of matrix integrity, likely influenced by lipid oxidation and/or starch–protein–lipid interaction. These effects have been linked to the formation of starch–lipid complexes during frying, which modify starch solubility and water diffusion behavior [67].

The DG in the noodle samples gradually increased across all samples, with TD showing the highest DG and TF the lowest (Figure 5F). This trend was consistent with that of their control counterparts. Greater gelatinization is desirable for non-fried noodles because it compensates for the absence of a porous frying structure by enhancing water absorption during the reconstitution process [68]. The lower DG in fried noodles is primarily attributed to the presence of lipid-starch complexes, which may form during frying and alter thermal transitions owing to restricted access to water [69]. Lower TF values than those of CF further suggest that endogenous fish lipids reinforce these interactions. Wang et al. [70] indicates that the interaction of lipids with starch and protein occurs during food production that involves intensive mixing in the presence of water, facilitating lipid bindings into a starch-lipid-protein system. Conversely, TD rising above CD at later storage stages indicates that in tray-dried systems, the fish-enriched matrix may evolve to support higher apparent gelatinization, possibly owing to moisture redistribution and structural relaxation [68]. This is consistent with the observed convergence of the rehydration ratios of the samples during storage.

### 3.4. Texture Profile Analysis (TPA)

**Figure 6:**
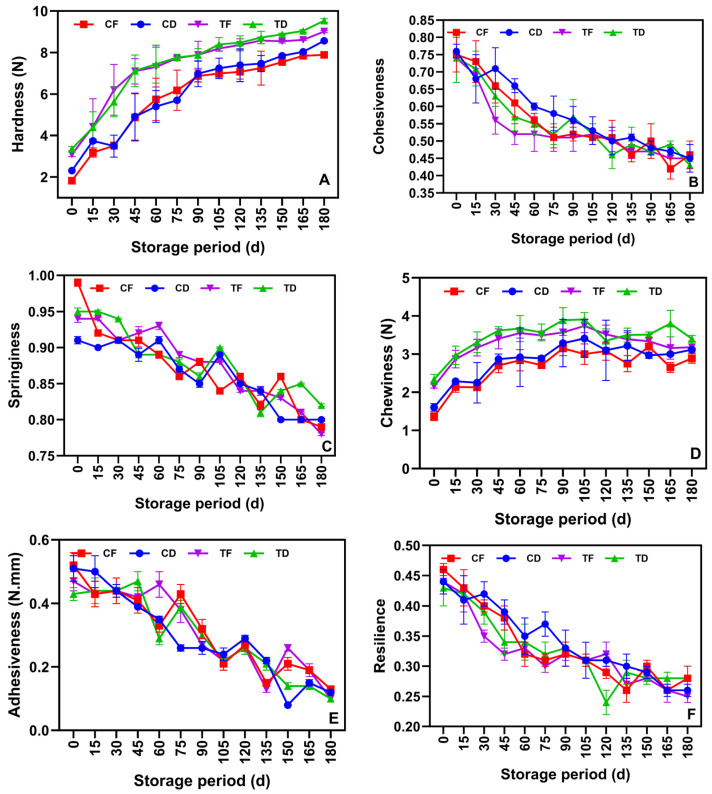
Changes in hardness (**A**), cohesiveness (**B**), springiness (**C**), chewiness (**D**), adhesiveness (**E**), and resilience (**F**) of instant noodles with or without CFP incorporation, processed by tray drying or deep frying, and stored under prolonged ambient conditions in M-LDPE packaging.

The TPA results for the tested instant noodle samples stored under ambient conditions are presented in Figure 6A–F. Across all treatments, the TPA response was significantly affected by the storage period, with strong influences from the formulation and drying method. The hardness of the samples progressively increased during prolonged storage (Figure 6A). CFP-incorporated samples, such as TD and TF, remained higher in hardness than CD and CF, indicating that CFP could enhance matrix densification, followed by the amplification of compression resistance during storage. This finding is consistent with the low-moisture starch-based food matrix, which exhibits a gradual development of a rigid structure owing to retrogradation and physicochemical relaxation during storage [71]. Compared with the deep-fried samples, the tray-dried samples exhibited higher hardness, which is mechanically consistent with foods with lower lipid fractions and a denser continuous phase. Deep-fried noodles generally retain more residual oil and have porous structures, which plasticize or cushion the noodle matrix during deformation [3,6]. Scott and Awika [55] reported that cereal-based dried foods progressively recrystallize the starch fractions and restructure the starch-protein interfaces, contributing to increased fracture and compression forces under prolonged storage. The cohesiveness of the test noodle samples is shown in Figure 6B. The results showed that prolonged storage significantly decreased the cohesiveness of the noodles, indicating a reduced ability of the noodle structure to maintain internal bonding under compression. In general, prolonged storage of dry foods tends to develop more brittle and weaker integrated matrix structures due to retrogradation-induced stiffening and weakening of the network integrity under continuous strain [72]. The TD and TF samples exhibited slightly lower cohesiveness than the CD and CF samples, owing to the partial replacement of WF with CFP, which reduced the gluten content, adversely affecting the continuity and elasticity of the noodle structure [63]. Similarly, the springiness of the noodle samples tended to decline with prolonged storage (Figure 6C). This indicates a reduced recovery of elasticity of the noodles from deformation and that prolonged storage progressively shifted the noodle samples to a less resilient mechanical response. This is related to moisture migration within the packaged noodles and the concomitant hardening, which could collectively reduce the tendency of the structure to rebound after compression [73]. In contrast, the gumminess of the tested samples increased during early storage (Figure 6D) and then approached a plateau, which is expected as gumminess is derived from hardness and cohesiveness [74]. When hardness is dominant, gumminess increases despite a decrease in the cohesiveness of the noodle samples. This pattern suggests that the stiffness of the noodle samples increased during storage, indicating the influence of composite parameters rather than the concurrent reduction in the internal bonding strength of the tested samples. The adhesiveness of the tested noodle samples gradually decreased during storage (Figure 6E). The reduction in adhesiveness indicates that prolonged storage reduced the surface stickiness of the noodle samples during compression. Studies have reported that a decline in adhesiveness is consistent with the reduced free or weakly bound water content on the surface of the noodles, as well as the formation of rigid starch-protein structures that limit tack development during probe withdrawal [75]. However, the difference in adhesiveness among the samples was minimal, implying that under M-LDPE packaging and ambient storage conditions, the surface adhesion was less sensitive to CFP incorporation and drying method than bulk structuring parameters such as hardness and cohesiveness. Furthermore, the resilience results exhibited a consistent decline in all tested samples (Figure 6F), indicating that prolonged storage reduced the overall elasticity and tendency to recover during prolonged storage. Among the samples, CF and TF samples exhibited a slightly faster decline in resilience level compared to CD and TD. This is because lipid-rich systems are more susceptible to oxidative deterioration, potentially altering the matrix flexibility and interfacial interaction of the samples [76]. Overall, the TPA results indicated that prolonged ambient storage promoted progressive hardening in the noodle samples, followed by reduced elasticity and recovery, which is characteristic of low-moisture noodle systems. The tray-dried samples exhibited more pronounced stiffness development, and the fried samples retained a comparatively softer deformation behavior owing to the residual oil and porosity effects.

### 3.5. Proximate Composition

**Figure 7:**
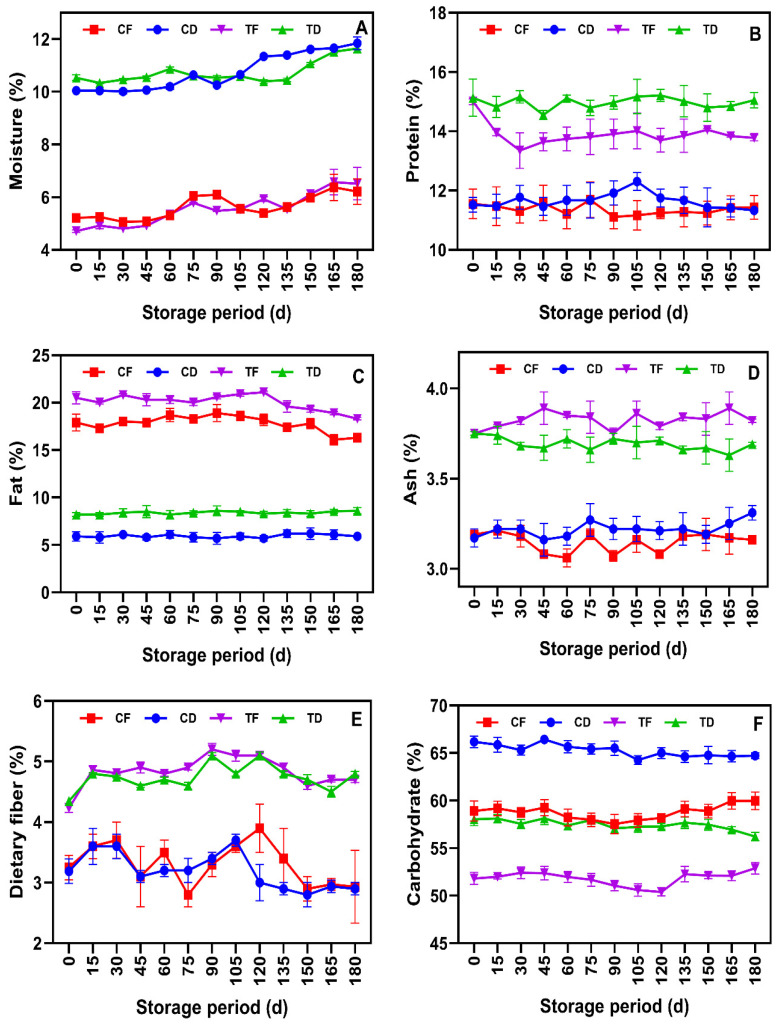
Changes in moisture (**A**), protein (**B**), fat (**C**), ash (**D**), dietary fiber (**E**), and carbohydrate contents (**F**) of instant noodles with or without CFP incorporation, processed by tray drying or deep frying, and stored under prolonged ambient conditions in M-LDPE packaging.

The proximate compositions of the tested noodle samples are shown in Figure 7A–F. Overall, the results showed that prolonged storage caused gradual treatment-dependent changes in the proximate composition of the tested samples, rather than abrupt shifts during ambient storage in M-LDPE packaging. The moisture content of the tested samples exhibited a progressive increase throughout storage, with a clear time-dependent trend (Figure 7A). At the beginning of storage, CF and TF samples had significantly lower moisture levels (4.0–4.5%) than CD and TD samples (10–11%), which was consistent with the expected outcome of the drying methods. Gulia et al. [21] observed a similar moisture range for fried (2–5%) and air-dried (8–12%) instant noodles. Frying promotes rapid surface evaporation and internal vapor generation under high heat flux, whereas tray drying relies on slower, diffusion-controlled dehydration, resulting in higher residual moisture [77]. Prolonged storage increased the moisture content in the samples by about 1–2%, likely due to gradual sorption and equilibrium with ambient humidity. Though the M-LDPE packages suppress the moisture increment, they do not completely prevent it, as water vapor ingresses through seals and interfaces [60]. Protein content in the noodle samples was primarily influenced by the formulation rather than the drying methods (Figure 7B). TF and TD samples, which were incorporated with CFP, consistently maintained higher protein levels (14–16%) than the CF and CD samples (11–12%). Throughout the storage, the protein content in the samples remained stable, indicating the persistence of the CFP fortification effect. This finding is in agreement with prior studies that report the enhancement and stabilization of protein levels found in the fish-based fortification in convenience food during storage [8,10,78]. The results of fat content in the tested instant noodle samples are shown in Figure 7C. Overall, the trend showed that fat content was strongly affected by the tested drying methods (particularly in TF and CF samples) at baseline and continued to vary throughout the storage period. In general, deep fat frying increases the fat content in food products due to oil absorption via capillary penetration and partial replacement of water during moisture loss, thus leading to a porous structure [79].

CFP incorporation in the noodles elevated the fat content owing to its intrinsic fish lipid content. During prolonged storage, the noodle samples exhibited a gradual decrease in fat content, especially in the fried samples, while the tray-dried samples remained relatively stable. This reduction in fat is likely due to lipid peroxidation and polymerization reactions that could possibly decrease the extractable fat fractions, even under protective packaging [67,80]. Ash content in the tested noodle samples remained stable throughout the storage period (Figure 7D). In comparison with control samples, the TF and TD samples exhibited consistently higher ash contents, reflecting the mineral contribution from CFP and dilution of WF. This is in accordance with the study of Vitorino et al. [81], who reported that inclusion of tilapia, salmon and tuna-based protein concentrate at a level of 10% in convenient food significantly increased ash content, which is likely due to the incorporation of natural minerals. Minor variations in the noodle samples were likely due to analytical variability and moisture normalization effects rather than storage-induced changes. Dietary fiber in the tested noodle samples showed greater variability than other proximate parameters (Figure 7E) and is likely due to analytical sensitivity and measurement dispersion of the methods rather than true compositional changes [82,83]. Carbohydrate levels in the noodle samples exhibited inverse patterns to other proximates, particularly protein, fat, and ash, with minor fluctuations that corresponded to moisture and lipid levels (Figure 7F). CD and CF samples maintained higher levels of carbohydrate, while CFP-fortified noodles were lower due to their higher protein, lipid, and mineral contents. These carbohydrate shifts in the samples during storage are likely due to compositional recalculations rather than starch degradation. Overall, prolonged storage caused modest and predictable changes in the proximate composition of the noodle samples. The M-LDPE packaging effectively limited moisture uptake and oxidative degradation and helped maintain the nutritional stability of both fried and tray-dried noodle samples.

### 3.6. Lipid Oxidation Indices

**Figure 8:**
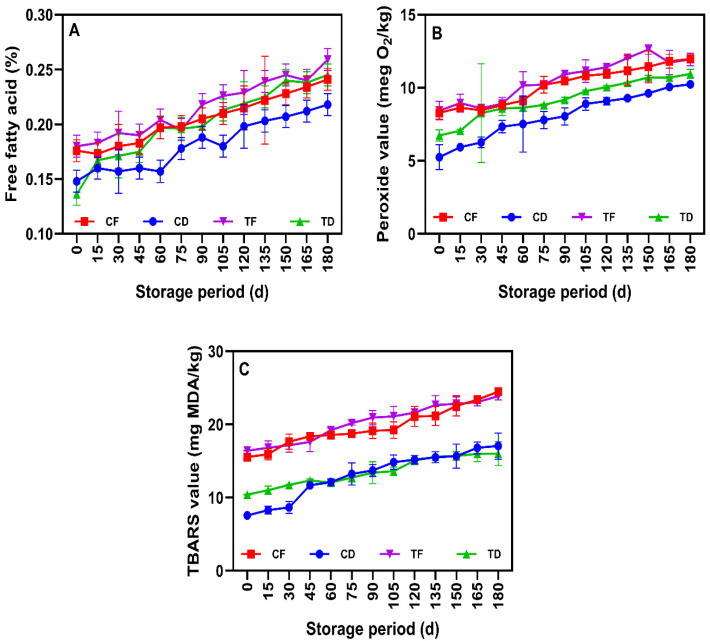
Changes in free fatty acid content (**A**), peroxide value (**B**), and TBARS value (**C**) of instant noodles with or without CFP incorporation, processed by tray drying or deep frying, and stored under prolonged ambient conditions in M-LDPE packaging.

The chemical stability of lipids plays a crucial role in analyzing the shelf life of instant noodles during storage, as the oxidation of unsaturated fatty acids could accelerate off-flavors, nutritional loss, and the formation of undesirable compounds [21]. The present study exhibited a progression of lipid deterioration, especially in the CFP-added instant noodle samples, despite the variation in drying methods during prolonged ambient storage (Figure 8). Overall, storage time was the dominant driver of lipid oxidation, as reflected by the progressive increases in primary and secondary oxidation indices such as PV and TBARS over the 180-day period, while formulation and dehydration method modulated the magnitude of these changes among treatments. FFA content serves as a hydrolytic rancidity indicator that occurs when triglycerides are hydrolyzed to form FFA by moisture and/or lipase activity. Throughout the storage, the FFA content in the noodle samples was relatively low and stable, ranging between 0.13 and 0.27% (Figure 8A). This indicates that the hydrolytic degradation of lipids in the noodles was limited during storage, and the low-moisture packaged format likely helped constrain hydrolysis rather than eliminating it entirely. This is evidenced by the lower water activity in the samples and controlled moisture increment, as they are required for enzymatic or chemical hydrolysis [84]. Among the processing methods, the deep-fried samples (CF and TF) generally showed slightly higher FFA levels than the tray-dried samples, particularly CD. These differences may be associated with oil exposure and thermal stress during frying, which can promote hydrolytic and oxidative changes.

The CFP-incorporated noodle samples tended to show slightly higher FFA than the WF-only control samples, which could be due to compositional differences introduced by CFP and greater susceptibility of CFP-associated lipids to hydrolytic changes during storage under low-moisture conditions, rather than differences in enzymatic activity [85]. Additionally, the final FFA values in the tested samples were maintained well below the critical limit of 0.5–0.8%, which is typically associated with a rancid flavor in fried snack foods [86], supporting the fact that hydrolytic rancidity was not the dominant contributor within the tested storage period. Consistent with this observation, the relatively stable FFA across storage suggests that oxidative pathways were more influential than hydrolysis in determining lipid deterioration during prolonged storage. PV represents the concentration of peroxides formed during storage [87], and the PV of the tested samples exhibited a continuous increase during storage (Figure 8B). The initial range of 5–8 meq O_2_ kg^−1^ fat increased to 10–13 meq O_2_ kg^−1^ fat at the end of storage. A consistent increase in PV in all samples occurred during storage, confirming that lipid autooxidation occurred in the processed food samples. The steady rise in PV from the early to late storage period indicates ongoing formation of hydroperoxides throughout storage rather than a transient oxidation event. Drying methods and CFP influenced PV development during storage. Among the samples tested, CD exhibited the lowest PV levels, while CF and TF showed higher PV and continued to remain elevated during storage. This could be due to the thermal oxidation of the frying oil and their relatively low a_w_, which may have contributed, as under low aw conditions the reduced hydration environment can facilitate oxidation reactions [76]. Overall, the CFP-incorporated noodle samples exhibited higher PV than the other samples. This could be attributed to the lipid profile of CFP, which is rich in PUFAs that are prone to oxidation and chain reactions [88]. Importantly, comparison at day 180 indicated that the fried treatments, particularly TF, remained among the highest PV levels, confirming that residual frying oil and PUFA-rich CFP jointly intensified primary oxidation during extended storage. Despite the elevated PV in the samples during storage, all samples were well below the PV limit reported for instant noodles (30 meq O_2_ kg^−1^ fat) [4]. This suggests that the M-LDPE package is likely to help limit oxygen and moisture transfer during storage, thereby maintaining acceptable oxidative stability throughout storage.

Similarly, the TBARS levels in the tested samples continuously increased during storage (Figure 8C), indicating progressive secondary lipid oxidation during ambient storage. In general, secondary oxidation products, particularly MDA and related aldehydes, are produced from the breakdown of hydroperoxides during the prolonged storage of instant noodles [89]. A gradual increase in TBARS values was observed in all samples, rising from an initial range of 8–16 mg MDA kg^−1^ to 17–24 mg MDA kg^−1^ at the end of storage. The parallel increase in both PV and TBARS indicates that active oxidation processes occurred during the storage of noodle samples. The concurrent increase further suggests that hydroperoxides formed during storage were progressively decomposed into secondary aldehydic products, consistent with advancing oxidation during late storage. Among all, CF and TF showed higher TBARS values, followed by tray-dried samples. This is due to the elevated temperature during frying, which initiates the decomposition of peroxides into secondary oxidative products, and continues until the end of storage [90]. Furthermore, similar to PV, TBARS was also high in the CFP-enriched fried noodles, particularly in the TF samples, and this is likely due to the combined effects of thermal stress and PUFAs. Comparison at day 180 confirmed that TF exhibited the greatest accumulation of secondary oxidation products, indicating poorer long-term oxidative stability among treatments, whereas CD remained comparatively lower, reflecting the combined benefit of lower initial lipid loading and milder thermal history. However, the secondary oxidation products in the tested samples were still within the typical range reported for stored instant noodles.

### 3.7. Microbial Quality

The microbiological stability of the instant noodle samples during ambient storage in metallized LDPE (M-LDPE) packaging was evaluated using total plate count (TPC), yeast and mold enumeration, and the presence or absence of *Escherichia coli*, *Salmonella* spp., and total coliforms, and the results are shown in Table 1. These indicators were selected in accordance with standard microbiological methods and notification of the Ministry of Industry: Industrial Product Standard for Instant Noodles [91]. During the first 150 days of storage, all noodle samples (CD, CF, TD, TF) remained below the detection limit for TPC, yeasts, and molds. This demonstrates that the combination of low water activity, dehydration processing, and metallized packaging effectively inhibited microbial proliferation in this low-moisture product [92]. The absence of detectable growth throughout most of the storage period suggests that the product matrix and packaging provided a hostile environment for microbial metabolism, consistent with previous findings on microbial stability in dehydrated noodle products. At the later storage intervals, limited bacterial growth was detected in selected treatments.

Specifically, TF showed measurable TPC at day 165 (3.79 ± 0.14 log_10_ CFU/g), increasing slightly by day 180 (4.84 ± 0.24 log_10_ CFU/g), while TD exhibited a lower TPC at day 180 (3.54 ± 0.17 log_10_ CFU/g). In contrast, CD and CF remained below the detection limit throughout the 180-day storage period. These values fall well within commonly accepted interpretive ranges for low-moisture cereal-based foods, where total aerobic counts up to 10^5^ CFU/g are considered microbiologically acceptable provided that pathogens and hygiene indicators remain absent [93]. The minor bacterial recovery at the terminal stage likely reflects gradual moisture equilibration or localized humidity exposure near the package seals during prolonged storage, rather than true spoilage activity. Yeasts and molds remained undetectable in all treatments at all storage intervals. This pattern is consistent with the known physiology of xerotolerant yeasts, which can sometimes grow at water activities as low as 0.65–0.70, whereas mold species generally require higher a_w_ (>0.80) [94]. The absence of molds across all treatments underscores the fungistatic effect of low a_w_ and high-temperature processing, as both frying and tray drying are known to inactivate mold spores effectively [95]. All samples tested negative for indicator and pathogenic microorganisms, including total coliforms, *E. coli*, and *Salmonella* spp., throughout the entire storage period. This confirms that the noodles were produced under good hygienic practices (GHP) and that the combination of heat treatment, dehydration, and hermetic packaging successfully prevented post-process contamination. These findings align with Codex Alimentarius microbiological standards, which stipulate the absence of *E. coli* and *Salmonella* spp. in ready-to-eat cereal-based foods and low-moisture products [96]. Overall, the results confirm that the instant noodle blocks maintained excellent microbiological quality and safety for 180 days of ambient storage under M-LDPE packaging. The occasional late-stage detection of low TPC in TF and TD did not exceed acceptable quality limits and is consistent with previous reports on the microbial stability of instant noodles and other low-moisture cereal-based products stored in barrier packaging [64,97]. This stability can be attributed to the combined effects of low water activity, limited nutrient accessibility, and restricted oxygen ingress, which collectively suppress microbial growth and ensure product safety during long-term storage [98].

### 3.8. Multivariate Relationship

**Figure 9:**
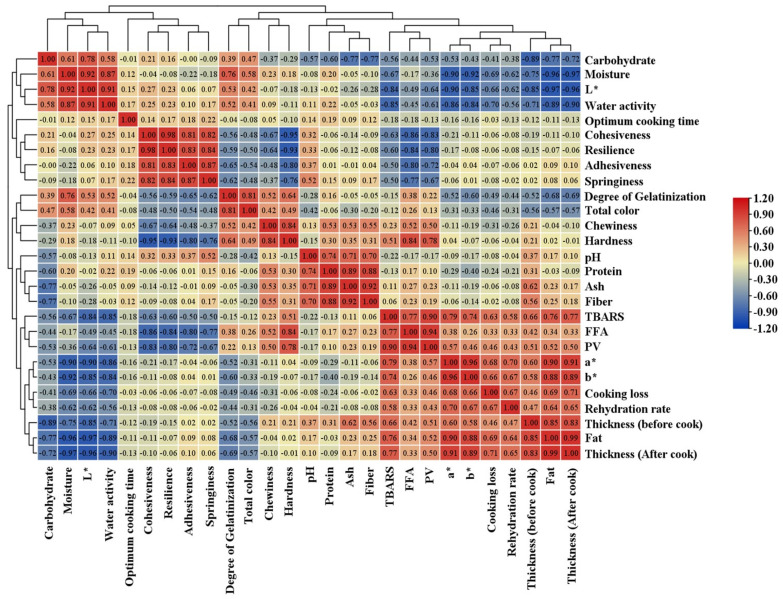
Pearson correlation heatmap with hierarchical clustering of the quality attributes of instant noodles with or without CFP incorporation, processed by tray drying or deep frying, and stored under prolonged ambient conditions in M-LDPE packaging.

Before multivariate modeling, the microbiological variables were screened for variance. Pathogenic bacteria (*Salmonella* spp. and *E. coli*) and hygiene indicators (coliforms, yeast, and molds) were not detected in any of the samples throughout the 180-day storage period (all results were below the limit of detection). Accordingly, these microbiological parameters were excluded from the PCA and correlation matrix analyses because they exhibited zero variance, and the inclusion of such data could be statistically uninformative and distort covariance-based computations. Correlation analysis revealed structured co-variation, particularly among rehydration, TPA, proximate composition, and lipid oxidation indicators (Figure 9). A significant rehydration and appearance block was observed, wherein moisture content demonstrated a strong correlation with L* (r = 0.92) and a_w_ (r = 0.87). In addition, the L* values exhibited a strong correlation with a_w_ (r = 0.91). The hydration-associated block contrasted with the fat-associated block, as evidenced by the strong negative correlations observed between fat and moisture (r = −0.96), fat and L* (r = −0.97), and fat and a_w_ (r = −0.89). The variables, particularly a* and b*, exhibited a strong positive correlation (r = 0.96) and were inversely associated with moisture content (a*: r = −0.90; b*: r = −0.92) and L* (a*: r = −0.90; b*: r = −0.85). The findings indicate that an increase in a* and b* is associated with a decrease in both L* and hydration levels. The rehydration rate, cooking loss, and post-cooking thickness were associated with the fat-related domain in the correlation structure. This association suggests that samples with higher lipid availability exhibited greater structural expansion during cooking and increased solute loss, which aligns with the directionality of multivariate separation observed. The texture attributes were categorized into two distinct clusters. Cohesiveness was strongly correlated with resilience (r = 0.98) and exhibited positive associations with springiness (r = 0.82) and adhesiveness (r = 0.81). This suggests coordinated alterations in the textural properties related to elasticity and integrity. Conversely, hardness was aligned with chewiness (r = 0.84) and showed strong negative correlations with cohesiveness (r = −0.95) and resilience (r = −0.93), indicating that increases in firmness and the force required for chewing were associated with diminished elastic recovery and matrix integrity, respectively.

**Figure 10:**
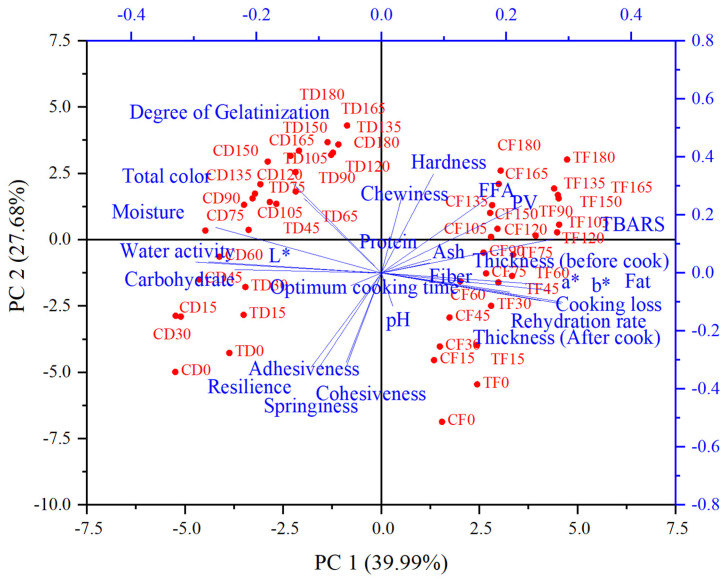
PCA of the quality attributes of instant noodles with or without CFP incorporation, processed by tray drying or deep frying, and stored under prolonged ambient conditions in M-LDPE packaging.

Correlation analysis revealed a proximate composition block characterized by strong intercorrelations among protein, ash, and fiber (protein–ash: r = 0.89; protein–fiber: r = 0.88; ash–fiber: r = 0.92). Additionally, pH was positively correlated with these variables (pH–protein: r = 0.74; pH–ash: r = 0.71; pH–fiber: r = 0.70). Furthermore, the oxidative indices revealed a distinct clustering pattern, with significant correlations observed among PV, TBARS, and FFA (PV–FFA: r = 0.94; PV–TBARS: r = 0.90; TBARS–FFA: r = 0.77). These oxidation markers were positively associated with fat content (fat–TBARS: r = 0.76; fat–PV: r = 0.52) and color changes (TBARS–a*: r = 0.79; TBARS–b*: r = 0.74), highlighting the critical role of lipid availability in promoting the formation of primary and secondary oxidation products during storage. PCA effectively summarized the multivariate structure, with the first two principal components accounting for 67.67% of the variance (PC1: 39.99%; PC2: 27.68%) (Figure 10). PC1 represented the primary distinction between the hydration and L* domains and the fat and oxidation domains. The variables loaded in the positive PC1 direction included fat, thickness (pre- and post-cooking), oxidative indices (TBARS, PV, and FFA), and color shift (a* and b*). The variables related to cooking performance, namely rehydration rate, cooking loss, and post-cooking thickness, were aligned in the same principal component 1 (PC1) direction as the fat and oxidation indices. This alignment indicates that the differentiation of the fried treatments was influenced not only by lipid oxidation behavior but also by structural responses associated with cooking. Conversely, the variables loading in the negative PC1 direction included moisture, a_w_, carbohydrate, and L* values, confirming the inverse relationship between hydration-related attributes and fat-associated oxidative behavior observed in the correlation matrix. PC2 primarily captured the TPA directionality. Hardness and chewiness were projected toward the upper region, whereas cohesiveness, resilience, adhesiveness, and springiness were projected toward the lower region, indicating that PC2 differentiated firmness-related changes from elasticity-related changes. In the score space, CD and TD samples were predominantly positioned on the negative side of PC1, consistent with their association with higher moisture, water activity, and L* values. In contrast, the CF and TF samples were predominantly positioned toward the positive side of PC1, consistent with their higher fat content and stronger alignment with the oxidation indices and color shift. With storage progression, fried samples exhibited greater displacement toward the oxidation-associated region (positive PC1), whereas tray-dried samples remained closer to the hydration-associated domain and exhibited a comparatively smaller multivariate drift. Overall, the combined correlation and PCA outcomes indicated that the drying method was the primary driver of multivariate separation, whereas storage time amplified divergence most clearly along the fat oxidation and associated color shift axis, particularly in the fried treatments.

## 4. Conclusions

This study investigated the effects of deep-fat frying and tray drying on the physicochemical, structural, and microbiological properties of instant noodles with and without CFP during prolonged ambient storage. Fried noodles exhibited a porous, blistered structure and a golden-yellow to reddish-brown appearance, whereas tray-dried noodles showed a denser structure and paler appearance. CFP incorporation increased protein, fat, and ash contents while reducing carbohydrate content, and it also darkened the noodles and modified pore geometry. Storage time induced progressive quality changes across all treatments, including gradual color drift and a continuous increase in lipid oxidation indices, with oxidation generally more pronounced in CFP-containing and fried noodles. Cooking-related attributes showed storage-dependent shifts, reflecting structural evolution of the noodle matrix during aging. Microbiological quality remained acceptable throughout the storage period. Although flavor attributes were not directly assessed in the present study, the progressive increases in lipid oxidation indices, particularly TBARS and peroxide values, observed across all treatments suggest a potential for storage-related off-flavor development, given the well-established relationship between secondary lipid oxidation products and rancid flavor perception in lipid-rich cereal-based foods. CFP-containing fried noodles showed the highest oxidation levels by day 180, indicating they may be most susceptible to flavor deterioration during prolonged storage. Future studies should incorporate sensory evaluation alongside volatile profiling to directly assess the flavor implications of the oxidative and Maillard-related changes observed in CFP-fortified instant noodles during ambient storage. Overall, the results demonstrate that the dehydration method and CFP fortification jointly govern long-term quality trajectories of fortified instant noodles under ambient storage, providing practical guidance for selecting processing conditions for nutritionally enhanced, shelf-stable products.

## Figures and Tables

**Table 1:** Microbiological quality of the instant noodles with or without CFP incorporation, processed by tray drying or deep frying, and stored under prolonged ambient conditions in M-LDPE packaging.

Storage Period	Total Plate Count (log_10_ CFU/g)				Total Yeast Count (log_10_ CFU/g)				Total Mold Count (log_10_ CFU/g)				Total Coliforms (MPN/g)				*Salmonella* (Presence/Absence, 25 g Sample)				*E. coli* (Presence/Absence in 25 g Sample)			
	CD	CF	TD	TF	CD	CF	TD	TF	CD	CF	TD	TF	CD	CF	TD	TF	CD	CF	TD	TF	CD	CF	TD	TF
**0**	ND	ND	ND	ND	ND	ND	ND	ND	ND	ND	ND	ND	ND	ND	ND	ND	ND	ND	ND	ND	ND	ND	ND	ND
**15**	ND	ND	ND	ND	ND	ND	ND	ND	ND	ND	ND	ND	ND	ND	ND	ND	ND	ND	ND	ND	ND	ND	ND	ND
**30**	ND	ND	ND	ND	ND	ND	ND	ND	ND	ND	ND	ND	ND	ND	ND	ND	ND	ND	ND	ND	ND	ND	ND	ND
**45**	ND	ND	ND	ND	ND	ND	ND	ND	ND	ND	ND	ND	ND	ND	ND	ND	ND	ND	ND	ND	ND	ND	ND	ND
**60**	ND	ND	ND	ND	ND	ND	ND	ND	ND	ND	ND	ND	ND	ND	ND	ND	ND	ND	ND	ND	ND	ND	ND	ND
**75**	ND	ND	ND	ND	ND	ND	ND	ND	ND	ND	ND	ND	ND	ND	ND	ND	ND	ND	ND	ND	ND	ND	ND	ND
**90**	ND	ND	ND	ND	ND	ND	ND	ND	ND	ND	ND	ND	ND	ND	ND	ND	ND	ND	ND	ND	ND	ND	ND	ND
**105**	ND	ND	ND	ND	ND	ND	ND	ND	ND	ND	ND	ND	ND	ND	ND	ND	ND	ND	ND	ND	ND	ND	ND	ND
**120**	ND	ND	ND	ND	ND	ND	ND	ND	ND	ND	ND	ND	ND	ND	ND	ND	ND	ND	ND	ND	ND	ND	ND	ND
**135**	ND	ND	ND	ND	ND	ND	ND	ND	ND	ND	ND	ND	ND	ND	ND	ND	ND	ND	ND	ND	ND	ND	ND	ND
**150**	ND	ND	ND	ND	ND	ND	ND	ND	ND	ND	ND	ND	ND	ND	ND	ND	ND	ND	ND	ND	ND	ND	ND	ND
**165**	ND	ND	ND	3.79 ± 0.14	ND	ND	ND	ND	ND	ND	ND	ND	ND	ND	ND	ND	ND	ND	ND	ND	ND	ND	ND	ND
**180**	ND	ND	3.54 ± 0.17	4.84 ± 0.24	ND	ND	ND	ND	ND	ND	ND	ND	ND	ND	ND	ND	ND	ND	ND	ND	ND	ND	ND	ND

**Note**: ND, not detected (below the method detection limit). Detection limits: total plate count and mold, <10 CFU/g; yeast, <100 CFU/g; total coliforms, <3.0 MPN/g. *Salmonella* and *E. coli* were not detected in 25 g of sample (presence/absence). Values are mean ± SD (*n* = 6) when detected.

## Data Availability

The original contributions presented in this study are included within the article. Further inquiries can be directed to the corresponding author.
